# Outcomes After Elective Inguinal Hernia Repair Performed by Associate Clinicians vs Medical Doctors in Sierra Leone: A Randomized Clinical Trial

**DOI:** 10.1001/jamanetworkopen.2020.32681

**Published:** 2021-01-11

**Authors:** Thomas Ashley, Hannah Ashley, Andreas Wladis, Håkon A. Bolkan, Alex J. van Duinen, Jessica H. Beard, Hertta Kalsi, Juuli Palmu, Pär Nordin, Kristina Holm, Michael Ohene-Yeboah, Jenny Löfgren

**Affiliations:** 1 Kamakwie Wesleyan Hospital, Kamakwie, Sierra Leone; 2 Department of General Surgery, North Cumbria University Hospital, Carlisle, United Kingdom; 3 The Lakes Medical Practice, Penrith, United Kingdom; 4 Department of Clinical and Experimental Medicine, Linköping University, Linköping, Sweden; 5 Department of Clinical and Molecular Medicine, Norwegian University of Science and Technology, Trondheim, Norway; 6 Clinic of Surgery, Trondheim University Hospital, Trondheim, Norway; 7 Lewis Katz School of Medicine, Temple University, Philadelphia, Pennsylvania; 8 Skåne University Hospital, Skåne, Sweden; 9 Kiruna Hospital, Kiruna, Sweden; 10 Department of Surgery and Perioperative Sciences, Umeå University, Umeå, Sweden; 11 Mälarsjukhuset, Eskilstuna, Sweden; 12 Department of Surgery, University of Ghana, Accra, Ghana; 13 Department of Molecular Medicine and Surgery, Karolinska Institutet, Karolinska University Hospital, Stockholm, Sweden

## Abstract

**Importance:**

Task sharing of surgical duties with medical doctors (MDs) without formal surgical training and associate clinicians (ACs; health care workers corresponding to an educational level between that of a nurse and an MD) is practiced to provide surgical services to people in low-resource settings. The safety and effectiveness of this has not been fully evaluated through a randomized clinical trial.

**Objective:**

To determine whether task sharing with MDs and ACs is safe and effective in mesh hernia repair in Sierra Leone.

**Design, Setting, and Participants:**

This single-blind, noninferiority randomized clinical trial included adult, healthy men with primary inguinal hernia randomized to receiving surgical treatment from an MD or an AC. In Sierra Leone, ACs practicing surgery have received 2 years of surgical training and completed a 1-year internship. The study was conducted between October 2017 and February 2019. Patients were followed up at 2 weeks and 1 year after operations. Observers were blinded to the study arm of the patients. The study was carried out in a first-level hospital in rural Sierra Leone. Data were analyzed from March to June 2019.

**Interventions:**

All patients received an open mesh inguinal hernia repair under local anesthesia. The control group underwent operations performed by MDs, and the intervention group underwent operations performed by ACs.

**Main Outcomes and Measures:**

The primary end point was hernia recurrence at 1 year. Outcomes were assessed by blinded observers at 2 weeks and 1 year after operations.

**Results:**

A total of 230 patients were recruited (mean [SD] age, 43.0 [13.5] years), and all but 1 patient underwent inguinal hernia repair between October 23, 2017, and February 2, 2018, performed by 5 MDs and 6 ACs. A total of 114 patients were operated on by MDs, and 115 patients were operated on by ACs. There were no crossovers between the study arms. The follow-up rate was 100% at 2 weeks and 94.1% at 1 year. At 1 year, hernia recurrence occurred in 7 patients (6.9%) operated on by MDs and 1 patient (0.9%) operated on by ACs (absolute difference, −6.0 [95% CI, −11.2 to 0.7] percentage points; *P* < .001).

**Conclusions and Relevance:**

These findings demonstrate that task sharing of elective mesh inguinal hernia repair with ACs was safe and effective. The task sharing debate should progress to focus on optimizing surgical training programs for nonsurgeons and building capacity for elective surgical care in low- and middle-income countries.

**Trial Registration:**

isrctn.org Identifier: ISRCTN63478884

## Key Points

**Question:**

Is groin hernia repair task sharing between medical doctors (MDs) and associate clinicians (ACs; health care workers corresponding to an educational level between that of a nurse and an MD) in Sierra Leone safe and effective?

**Findings:**

In this randomized clinical trial including 230 patients with primary inguinal hernia, 1-year postoperative hernia recurrence rates were 0.9% for patients operated on by ACs vs 6.9% for patients operated on by MDs, a statistically significant difference. The study shows noninferiority of ACs vs MDs.

**Meaning:**

These findings demonstrate that surgical task sharing to ACs in groin hernia surgical procedures was safe and effective and could be expanded to address the immense burden of disease owing to groin hernia.

## Teaser

This randomized clinical trial examined outcomes of inguinal hernia surgery performed by associate clinicians vs nonspecialist medical doctors among men in Sierra Leone.

## Introduction

Surgical conditions account for a large burden of disease, but 5 billion people do not have access to timely, high-quality surgical services at an affordable cost.^1^ Capacity to meet the immense need for surgical care remains very limited. The human resource crisis includes a pronounced scarcity of specialist surgeons in sub-Saharan Africa.^1^ In 2015, the 68th World Health Assembly^2^ passed a resolution on emergency and essential surgical and anesthesia care as an important part of universal health coverage. Task sharing was expressed as a part of the solution to the human resource shortage in surgery.^2^ Task sharing is the pragmatic sharing of clinical tasks among specialist and nonspecialist medical doctors (MDs) and midlevel health care workers, commonly called *associate clinicians* (ACs), who have an educational level between that of a nurse and an MD.^3,4^ The safety and effectiveness of surgical task sharing with ACs has been previously demonstrated in obstetric surgery.^5,6,7,8^ Surgery performed by MDs is often considered standard care, but very little research on task sharing with MDs exists.^9^ The level of evidence to support task sharing in general surgery is very limited.

This study was conducted in Sierra Leone, a small, low-income country in West Africa with a population of 7.4 million people in 2016.^10^ It ranks 184 of 189 countries on the Human Development Index.^11^ There is less than 1 surgeon per 100 000 population, and surgical treatment is mostly performed by MDs or ACs in large parts of the country.^12^ MDs receive basic surgical training during their 2-year internship after medical school; thereafter, they perform hernia repairs, cesarean sections, and other surgical procedures independently. ACs participate in a 3-year surgical training program organized since 2011 by CapaCare, a Norwegian nongovernmental organization, in collaboration with the Sierra Leone Ministry of Health and Sanitation and the United Nations Population Fund. This training program is also available for MDs, but very few MDs have enrolled.^13^

Groin hernia affects more than 200 million people worldwide and groin hernia repair is the most commonly performed general surgical procedure in sub-Saharan Africa.^14,15,16,17,18^ Therefore, it is a suitable condition for the evaluation of safety and effectiveness of task sharing in general surgery. A recent prospective cohort study from Ghana^19^ reported that task sharing of inguinal hernia repair with mesh among surgeons and MDs was safe and effective. The outcomes of ACs performing inguinal hernia repair have been assessed in Sierra Leone^13^ and Tanzania,^20^ but owing to the retrospective study design and short-term follow-up, the results were not conclusive. The objective of this randomized clinical trial was to compare outcomes after elective mesh inguinal repair in men performed by ACs vs MDs. MDs were chosen as the comparison group, because MDs represent standard care in Sierra Leone.

## Methods

### Study Design

This is a single blind, parallel, noninferiority randomized clinical trial comparing outcomes after primary inguinal hernia repair with mesh in adult men in Sierra Leone performed by ACs vs MDs. Ethical approval was granted by the Sierra Leone Ethics and Scientific Review Committee. Participants provided written or thumb-printed informed consent prior to inclusion in the study. The study adhered to the Consolidated Standards of Reporting Trials (CONSORT) reporting guideline, and no alterations were made to the study design during the study period (Trial Protocol in Supplement 1). It was hypothesized that the ACs would be noninferior to the MDs in terms of hernia recurrence at 1 year.

The study was carried out at Kamakwie Wesleyan Hospital, located in the Northern Province of Sierra Leone. This first-level (district) hospital has 110 beds and provides basic services in pediatrics, gynecology and obstetrics, internal medicine, and surgery for an entirely rural catchment population of approximately 200 000 people.

### Participants

Men aged 18 years or older with primary reducible inguinal hernia who had American Society of Anesthesiologists (ASA) scores of 1 or 2 (of 6) were eligible to participate, provided they were able to give informed consent. The exclusion criteria were obvious substance abuse and known or suspected coagulopathy. Recruitment was conducted through radio announcements and with assistance from local leadership.

The surgical procedures were provided to participants at no cost. Participants received a transport refund of US $6.50 per visit to the hospital.

### Randomization and Masking

The randomization sequence was generated online by one of us (J.L.) using blocks of 4, 6, and 8.^21^ The study number and randomization arm were written on identical pieces of papers and sealed in opaque envelopes. At the beginning of each operation day, the operating list was determined. Allocation to the control (MD) or intervention (AC) arm was performed by a study coordinator who opened the envelopes when the next patient on the list was taken to the operating room. The study coordinator did not participate in the operations or the generation of the operating list.

Patient follow-up, including assessment of the study end points, was performed by 3 blinded MDs who did not participate in the operations (T.A., H.A., and J.L.). One of these observers was a surgeon (T.A.), and he reviewed all patients.

### Procedures

Anterior mesh inguinal hernia repair is associated with an almost 50% decreased risk of recurrence compared with tissue techniques.^22^ In this study, anterior mesh inguinal hernia repair was performed under local anesthesia according to the procedure described by Lichtenstein et al.^23,24^ The local anesthesia was a mix of 20 mL of lidocaine (10 mg/mL) with 20 mL of bupivacaine (5 mg/mL). Conversion to general anesthesia was possible if needed. A light-weight commercial polypropylene mesh was used for all patients. The patients received 1 dose of 1.5 g oral flucloxacillin 1 to 4 hours prior to the operation. All patients were observed for at least 2 hours postoperatively and were assessed by an MD before going home. Patients with concerns, complications, or late operations were discharged the next day. All patients received postoperative analgesia: 50 mg of diclofenac 2 times daily and 1g of paracetamol 4 times daily for 5 days.

Participants in the control group were operated on by MDs, while participants in the intervention group were operated on by ACs. The MDs had all completed medical school and 2 years of internship, which included rotations in general surgery and obstetrics and gynecology at first-level hospitals. The ACs had participated in the CapaCare surgical training program.^15^ Candidates with a diploma in community health (a 3-year training program) and with an additional minimum of 2 years of work experience as community health officers are eligible to apply for the CapaCare program. The duration of the program is 3 years, including 1 year of internship. All MDs and ACs were routinely performing hernia repair using tissue techniques as part of their current jobs. They had not been trained to perform mesh repair prior to their participation in the study. eTable 1 in Supplement 2 provides further information about the ACs and MDs.

Before patients were included in the study, the MDs and the ACs participated in an inguinal hernia mesh repair training program designed for a similar study in Ghana.^19^ The training consisted of a 1-day theoretical module and a hands-on module of supervised surgeries that lasted 1 to 3 days, depending on the need of each trainee to reach proficiency. The surgical practitioners’ skills were assessed by senior consultant surgeons from Sweden, Norway, Lithuania, and Ghana using the Groin Hernia Operative Performance Rating System by the American College of Surgeons,^25^ which grades the competence of the surgical practitioner in all these steps of a hernia repair on a scale of 1 to 5, with high score indicating better performance. To qualify to operate on patients in this study, the trainees needed to achieve a 4 or higher in the overall performance score.

Data were collected on 4 separate occasions. Preoperative data included patient characteristics, self-assessed health score (range, 0-10, with higher scores indicating better health), and symptoms from the hernia. Pain was assessed using the Inguinal Pain Questionnaire (IPQ), which assesses the worst pain during the past week. This 7-degree scale has been validated for the assessment of pain after groin hernia repair.^26^ The 7 levels range from 1 (ie, no pain) to 7 (ie, severe pain that requires immediate medical attention). Physical examination was carried out to verify the diagnosis and to determine the ASA score. Perioperative data collection occurred during and immediately after the operation and included information regarding the surgical procedure. Postoperative data collection occurred at 2 weeks and 1 year after the operation and included an interview and physical examination focusing on the study outcome measures.

### Outcomes

The primary outcome of the study was hernia recurrence within 1 year after the operation. Recurrence was defined as a palpable mass with cough impulse on the same side as the repair. Secondary outcomes recorded 2 weeks after the surgery included postoperative complications (ie, hematoma, more pain than expected, infection, seroma, impaired wound healing, and urinary retention). Secondary outcomes recorded at 1 year included chronic pain according to the IPQ, patient satisfaction, and self-assessed health scale.

### Statistical Analysis

The sample size of this noninferiority trial was determined using assumptions of 80% power, a significance level of 5%, and a noninferiority limit of 5 percentage points. The estimated recurrence rate in both groups was 2%. In a previous study examining mesh inguinal hernia repair carried out by the study team,^27^ the recurrence rate was less than 1%. In that 2016 study,^27^ the operations were performed by senior specialist surgeons; therefore, a higher recurrence rate was expected in this study. A noninferiority margin of 5% was deemed adequate, as a difference greater than this could have implications for policy and training. Based on these assumptions, the sample size was 97 participants in each group. Correcting for an expected loss to follow-up of 15%, the final sample size was 228 participants in total.

Continuous variables are presented as mean and SD, while binary variables are presented as numbers and percentages. For comparison between the 2 study arms, a χ^2^ test or Fisher exact test was used for binary outcomes and a 2-sample *t* tests was used for continuous variables. The 95% CIs of absolute difference between groups were calculated according to Jeffreys.^28^ Calculations were performed using Excel 2013 (Microsoft), SPSS statistical software version 24 (IBM) and Stata version 15.1 (StataCorp). All patients who underwent operations were included in the statistical analysis. Data were analyzed from March to June 2019.

## Results

A total of 230 patients were included and randomized to the control or the intervention group. One patient who had been enrolled in the study ultimately declined surgical treatment and was excluded from the analysis; therefore, a total of 114 patients underwent operations by MDs and 115 patients underwent operations by ACs. The mean (SD) age of the patients was 43.0 (13.5) years. The baseline characteristics of the patients were not significantly different between study groups (Table 1). Operations were performed during three 2-week periods between October 23, 2017, and February 2, 2018.

**Table 1.** Baseline Characteristics of Study Participants Stratified by Treating Practitioner

Characteristic	Participants, No. (%)	
	Associate clinician (n = 115)	Medical doctor (n = 114)
Age, mean (SD), y	43.7 (14.6)	42.3 (12.4)
BMI, mean (SD)	21.8 (4.8)	21.2 (2.3)^a^
ASA classification score 1^b^	52 (45.2)	51 (44.7)
Smoking status		
Current	47 (40.9)	54 (47.4)
Previous	21 (18.3)	15 (13.2)
Scrotal hernia	54 (48.6)	58 (51.8)
Size of scrotal hernia, mean (SD), cm^c^	14.4 (5.1)	14.1 (6.0)
IPQ score, mean (SD)^d^	2.9 (1.5)	3.1 (1.6)
Self-Assessed Health Score, mean (SD)^e^	76.8 (15.4)^a^	79.5 (13.7)^a^

Abbreviations: ASA, American Society of Anesthesiologists; BMI, body mass index (calculated as weight in kilograms divided by height in meters squared); IPQ, Inguinal Pain Questionnaire.

^a^ Includes data for 113 participants, as information was missing for 1 participant.

^b^ Range, 1 to 6, with lower score indicating fewer comorbidities and better health.

^c^ Measured from the pubic tubercle to the most distal part of the hernia.

^d^ Range, 1 to 7, with higher score indicating more severe pain.

^e^ Range, 0 to 100, with higher score indicating better health.

Data for primary and secondary end points were analyzed per protocol, as there was no crossover between study groups. One patient received ketamine anesthesia in addition to local anesthesia owing to inadequate pain control. After 2 weeks (mean [range], 14.8 [12-27] days), all 229 patients who had undergone surgical treatment were followed up. After 1 year (mean [range], 426.7 [377-492] days), 210 of 224 patients (93.8%) underwent follow-up interviews and physical examinations. At 1 year, 5 patients (2.2%) had died and 13 patients (5.8%) were lost to follow-up (Table 2; Figure).

**Table 2.** Recurrence and Death Among Study Participants at 1 Year Stratified by Treating Practitioner

Outcome	Participants, No./Total No. (%)		Absolute difference, percentage points (95% CI)	*P* value
	Associate clinician	Medical doctor		
Recurrence	1/109 (0.9)	7/102 (6.9)^a^	−6.0 (−11.2 to −0.7)	<.001^b^
Death	2/111 (1.8)	3/105 (2.9)	−1.0 (−5.1 to 3.0)	.68

^a^ Including one early recurrence.

^b^ Test of absolute difference in relation to the noninferiority limit of 5 percentage points.

**Figure.**
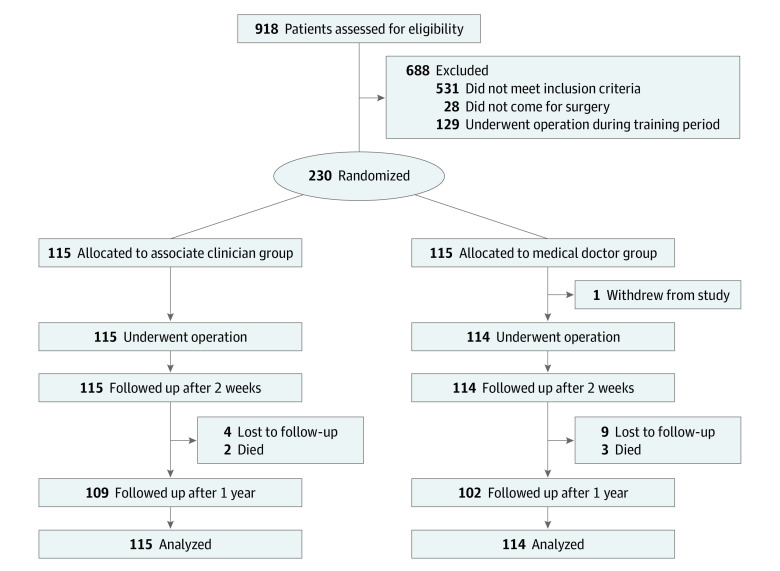
Patient Recruitment Flowchart All deaths occurred later than 30 days postoperatively.

After 1 year, 7 recurrences (6.9%) had been detected in the MD-treated group and 1 recurrence (0.9%) was identified in the AC-treated group (*P* = .02). The absolute difference between the MD and the AC groups was −6.0 (95% CI, −11.2% to −0.7%) percentage points (*P* < .001), thus demonstrating noninferiority of treatment by ACs compared with treatment by MDs (Table 3). Of 7 recurrences in the MD group, 1 was an early recurrence, which was treated with a second operation 5 days after the initial operation.

**Table 3.** Postoperative Complications at 2 Weeks Stratified by Treating Practitioner

Complication	No. (%)		Absolute difference, percentage points (95% CI)	*P* value
	Associate clinician, (n = 115)	Medical doctor (n = 114)		
Any	34 (29.6)	34 (29.8)	−0.2 (−12.1 to 11.6)	.97
Pain more than expected	6 (5.2)	5 (4.4)	0.8 (−4.7 to 6.4)	.77
Impaired wound healing	8 (7.9)	9 (7.9)	−0.9 (−7.7 to 5.9)	.34
Wound infection requiring antibiotics	6 (5.2)	4 (3.5)	1.7 (−3.6 to 7.0)	.75
Hematoma/seroma	17 (14.8)	20 (17.5)	−2.8 (−12.3 to 6.8)	.57
Reoperation	2 (1.7)	1 (0.9)	0.9 (−2.1 to 3.8)	>.99

A total of 3 patients (2.9%) in the MD-treated group and 2 patients (1.8%) in the AC-treated group died before the 1 year follow-up (Table 2). No patients died within 30 days of the operation. Details regarding the deaths were retrieved through interviews with hospital staff, family, or friends, and none were deemed likely due to the surgical operation (eTable 2 in Supplement 2).

The distribution of postoperative complications at 2 weeks was similar in the 2 groups. No statistically nor clinically significant differences were detected. The most common complication was hematoma or seroma on the side of the operation, occurring in 37 patients (16.2%). Overall, 10 patients (4.4%) developed infections requiring antibiotic treatment, 3 patients (1.3%) required a reoperation owing to early recurrence (1 patient), postoperative bleeding (1 patient), or drainage of infection (1 patient). After 1 year, all patients reported fewer symptoms from the groin than before the operation, and 208 patients (99.0%) were satisfied with the result of the operation (Table 4).

**Table 4.** Secondary End Points at 1 Year Stratified by Treating Practitioner

End point	Participants, No. (%)		Absolute difference, percentage points (95% CI)	*P* value
	Associate clinician, (n = 109)	Medical doctor, (n = 102)		
Fewer groin symptoms compared to before the surgery	109 (100)	102 (100)	0	>.90
Satisfied with the result	108 (99.1)	100 (99.0)^a^	0.1 (−2.6 to 2.7)	>.99
IPQ score, mean (SD)^b^	1.2 (0.5)	1.3 (0.9)	−0.1 (−0.3 to 0.1)	.24
1	94 (86.2)	87 (85.3)	0.9 (−8.5 to 10.4)	.69
2-3	14 (12.8)	12 (11.8)	1.1 (−7.8 to 9.9)	
≥4	1 (0.9)	3 (2.9)	−2.0 (−5.8 to 1.7)	
Self-assessed health status^c^	88.2 (12.4)^d^	88.9 (13.1)	−0.7 (−4.2 to 2.8)	.69
Difference vs baseline	10.5 (18.1)^e^	9.4 (18.7)^a^	1.1 (−4.0 to 6.1)	.68

Abbreviation: IPQ, Inguinal Pain Questionnaire (score: 1 = no pain to 7 = severe pain in need for immediate medical attention).

^a^ Information missing for 1 patient.

^b^ Range, 1 to 7, with higher score indicating more severe pain.

^c^ Range, 0 to 100, with higher score indicating better health.

^d^ Information missing for 2 patients.

^e^ Information missing for 4 patients.

## Discussion

In this randomized clinical trial, ACs in Sierra Leone were noninferior to MDs in terms of recurrence after mesh inguinal hernia repair 1 year postoperatively. No clinically nor statistically significant differences between patients operated on by MDs vs ACs were detected in terms of postoperative complications, chronic pain, patient satisfaction, and self-assessed health.

The recurrence rate among patients treated by ACs was similar to that of patients treated by MDs and specialist surgeons in Ghana (1.8%),^19^ and senior consultant surgeons in Uganda (0.7%).^27^ It also compares favorably with outcomes in very high–resource settings (recurrence rate, 0%-4.9%).^29^ The recurrence rate in the group operated on by MDs was surprisingly high, and the reasons for this remain unclear. In a study set in Ghana,^19^ MDs (recurrence rate, 0.9%) were found to be noninferior to specialist surgeons (recurrence rate, 2.8%). We theorize that the most likely reason is that the surgical training during medical school and internship in Sierra Leone are inadequate preparation for independent practice. Although MDs represent standard care in many countries, little is known about the surgical training that they receive and outcomes after the surgical procedures that they perform.^9^ A future study could investigate surgical training, supervision, and outcomes after surgery performed by MDs in a variety of low-resource countries. Setting-appropriate strategies to deliver state-of-the-art surgical training for all surgical practitioners could be developed based on the findings from such a study.

Surgical site infection remains an important safety concern.^30^ In this study, an overall infection rate of 4.4% was observed. In high-income countries, postoperative infection rates vary substantially (0%-15%).^31^ In previous research on mesh inguinal hernia repair in adult men, the reported infection rate was 3.4% in Uganda^27^ and 8.0% in Ghana.^19^ No deep infections occurred, and removal of the mesh was never required in our study or in the Ugandan^27^ and Ghanian^19^ studies. Therefore, it can be concluded that the use of mesh in elective inguinal hernia repair in men is safe in these 3 low-resource settings with pronounced hygienical challenges. As mesh repair is associated with a nearly halved risk of recurrence compared with tissue techniques, it should be routinely used also in low-resource settings.^22^

This study provides further evidence that local anesthesia and outpatient surgery for inguinal hernia is safe and well accepted in low-resource countries, such as Ghana,^19^ Uganda,^27^ and Sierra Leone. Local anesthesia in inguinal hernia repair is associated with significantly fewer postoperative complications than spinal and general anesthesia.^32^ It also has potential to spare the scarce anesthesia resources for other important tasks. Outpatient surgery for elective inguinal hernia repair as a routine could improve the effectiveness of service delivery and reduce costs for the patients, health care institutions, and societies.^33^

Elective surgery for groin hernia is associated with reduced risk of adverse outcomes compared with emergency surgery.^34^ Elective surgical services should be expanded to meet the vast unmet need for surgical treatment in sub-Saharan Africa. Further development and increase scale of the training program used for this study could spearhead a movement toward a system that caters for elective surgical care, not just emergencies. Research assessing the training program and trainer assessment using the Operative Performance Rating System scores is planned for this reason.

The unmet need for surgical procedures has been estimated at more than 143 million procedures year.^1^ Task sharing is part of the solution to meet this need.^2^ While MDs performing surgical procedures is considered standard care in many countries, task sharing with ACs remains a controversial topic.^35^ This study demonstrates the safety of task sharing with ACs in the delivery of an important surgical procedures, and this information is an important contribution to the task sharing discussion. The task sharing debate should progress to focus on how surgical training, supervision, and monitoring can be developed to ensure that all surgical practitioners can deliver high quality surgical services.

### Strengths and Limitations

The main strengths of this trial are the study design and the implementation of the study. Every aspect of the implementation of the study was strictly monitored by the lead researchers. The follow-up rates were very high, reducing the risk of selection bias. Therefore, the internal validity is high. The study hospital was typical for first-level hospitals in sub-Saharan Africa. Inguinal hernia repair is a common general surgical procedure worldwide. Inguinal hernia repair with mesh is a standard procedure that can be carried out in all health care systems given availability of mesh and skilled surgical practitioners. The study findings are therefore generalizable, with high external validity.

This study also has some limitations. The strict monitoring of each aspect of implementation of this study is not routine for surgical practice in general. The outcomes of task sharing in routine care remains to be evaluated. In day-to-day surgical practice, patient selection is different from the selection process in this study. Emergency hernia repair and repair of recurrent hernia and hernia in women and children are all part of routine surgical care. Future research should investigate the safety and effectiveness of task sharing in these instances. Recurrence after hernia repair can occur after 1 year. We have planned a 3-year follow-up to take place during 2021.

## Conclusions

This randomized clinical trial found that surgical task-sharing with ACs in elective groin hernia repair in men in Sierra Leone was safe and effective. Measures should be taken to ensure high-quality training, monitoring, and evaluation for all surgical practitioners. Groin hernia repair with mesh under local anesthesia was performed as outpatient surgery was safe and should be widely implemented to benefit other patients in low-resource settings.

## Notes

Supplement 1. **Trial Protocol**

Supplement 2. **eTable 1.** Trainee and Trainer Characteristics **eTable 2.** Summary of Mortalities at 1 Year

Supplement 3. **Data Sharing Statement**

## References

[1] Meara JG, Leather AJM, Hagander L, Global Surgery 2030: evidence and solutions for achieving health, welfare, and economic development. Lancet. 2015;386(9993):569-624. doi:10.1016/S0140-6736(15)60160-X 25924834

[2] World Health Assembly Strengthening emergency and essential surgical care and anaesthesia as a component of universal health coverage. Accessed July 15, 2020. https://apps.who.int/gb/ebwha/pdf_files/WHA68/A68_R15-en.pdf?ua=1

[3] World Health Organization Task Shifting: Global Recommendations and Guidelines. WHO Press; 2008.

[4] Federspiel F, Mukhopadhyay S, Milsom P, Scott JW, Riesel JN, Meara JG Global surgical and anaesthetic task shifting: a systematic literature review and survey. Lancet. 2015;385(suppl 2):S46. doi:10.1016/S0140-6736(15)60841-8 26313095

[5] van Duinen AJ, Kamara MM, Hagander L, Caesarean section performed by medical doctors and associate clinicians in Sierra Leone. Br J Surg. 2019;106(2):e129-e137. doi:10.1002/bjs.11076 30620069 PMC6590228

[6] Wilson A, Lissauer D, Thangaratinam S, Khan KS, MacArthur C, Coomarasamy A A comparison of clinical officers with medical doctors on outcomes of caesarean section in the developing world: meta-analysis of controlled studies. BMJ. 2011;342:d2600. doi:10.1136/bmj.d2600 21571914 PMC3272986

[7] Gessessew A, Barnabas GA, Prata N, Weidert K Task shifting and sharing in Tigray, Ethiopia, to achieve comprehensive emergency obstetric care. Int J Gynaecol Obstet. 2011;113(1):28-31. doi:10.1016/j.ijgo.2010.10.023 21315350

[8] Pereira C, Bugalho A, Bergström S, Vaz F, Cotiro M A comparative study of caesarean deliveries by assistant medical officers and obstetricians in Mozambique. Br J Obstet Gynaecol. 1996;103(6):508-512. doi:10.1111/j.1471-0528.1996.tb09797.x 8645640

[9] Falk R, Taylor R, Kornelsen J, Virk R Surgical task-sharing to non-specialist physicians in low-resource settings globally: a systematic review of the literature. World J Surg. 2020;44(5):1368-1386. doi:10.1007/s00268-019-05363-7 31915975

[10] Statistics Sierra Leone Sierra Leone 2015 Population and Housing Census: national analytical report. Accessed April 22, 2019. https://sierraleone.unfpa.org/sites/default/files/pub-pdf/National%20Analytical%20Report.pdf

[11] United Nations Development Programme Human development reports: Sierra Leone human development indicators. Accessed April 22, 2019. http://hdr.undp.org/en/countries/profiles/SLE

[12] Bolkan HA, Hagander L, von Schreeb J, The surgical workforce and surgical provider productivity in Sierra Leone: a countrywide inventory. World J Surg. 2016;40(6):1344-1351. doi:10.1007/s00268-016-3417-1 26822155 PMC4868859

[13] Bolkan HA, van Duinen A, Waalewijn B, Safety, productivity and predicted contribution of a surgical task-sharing programme in Sierra Leone. Br J Surg. 2017;104(10):1315-1326. doi:10.1002/bjs.10552 28783227 PMC5574034

[14] Beard JH, Ohene-Yeboah M, devries CR, Schecter WP Hernia and hydrocele In: Debas HT, Donkor P, Gawande A, Jamison DT, Kruk ME, Mock CN, eds. Essential Surgery: Disease Control Priorities. Third ed (volume 1) The International Bank for Reconstruction and Development/The World Bank; 2015. 26740991

[15] CapaCare Surgical training program: annual activity report 2017. Accessed April 22, 2019. https://capacare.org/wp-content/uploads/2016/12/CapaCare-Annual-Report-2017-3.pdf

[16] Bolkan HA, Hagander L, von Schreeb J, Who is performing surgery in low-income settings: a countrywide inventory of the surgical workforce distribution and scope of practice in Sierra Leone. Lancet. 2015;385(suppl 2):S44. doi:10.1016/S0140-6736(15)60839-X 26313093

[17] Galukande M, von Schreeb J, Wladis A, Essential surgery at the district hospital: a retrospective descriptive analysis in three African countries. PLoS Med. 2010;7(3):e1000243. doi:10.1371/journal.pmed.1000243 20231871 PMC2834708

[18] Löfgren J, Kadobera D, Forsberg BC, Mulowooza J, Wladis A, Nordin P District-level surgery in Uganda: indications, interventions and perioperative mortality. Surgery. 2015;158(1):7-16. doi:10.1016/j.surg.2015.03.022 25958070

[19] Beard J, Ohene-Yeboah M, Tabiri S, Outcomes after inguinal hernia repair with mesh performed by medical doctors and surgeons in Ghana. JAMA Surg. 2019;154(9):853-859. doi:10.1001/jamasurg.2019.1744 31241736 PMC6596328

[20] Beard JH, Oresanya LB, Akoko L, Mwanga A, Mkony CA, Dicker RA Surgical task-shifting in a low-resource setting: outcomes after major surgery performed by nonphysician clinicians in Tanzania. World J Surg. 2014;38(6):1398-1404. doi:10.1007/s00268-013-2446-2 24407941

[21] Sealed Envelope Create a blocked randomisation list. Accessed October 22, 2017. https://www.sealedenvelope.com/simple-randomiser/v1/lists

[22] Lockhart K, Dunn D, Teo S, Mesh versus non-mesh for inguinal and femoral hernia repair. Cochrane Database Syst Rev. 2018;9:CD011517. doi:10.1002/14651858.CD011517.pub2 30209805 PMC6513260

[23] Lichtenstein IL, Shulman AG, Amid PK, Montllor MM The tension-free hernioplasty. Am J Surg. 1989;157(2):188-193. doi:10.1016/0002-9610(89)90526-6 2916733

[24] Amid PK, Shulman AG, Lichtenstein IL Local anesthesia for inguinal hernia repair step-by-step procedure. Ann Surg. 1994;220(6):735-737. doi:10.1097/00000658-199412000-00004 7986138 PMC1234473

[25] American Board of Surgery Resident assessments. Accessed April 28, 2019. https://www.absurgery.org/default.jsp?certgsqe_resassess

[26] Fränneby U, Gunnarsson U, Andersson M, Validation of an Inguinal Pain Questionnaire for assessment of chronic pain after groin hernia repair. Br J Surg. 2008;95(4):488-493. doi:10.1002/bjs.6014 18161900

[27] Löfgren J, Nordin P, Ibingira C, Matovu A, Galiwango E, Wladis A A randomized trial of low-cost mesh in groin hernia repair. N Engl J Med. 2016;374(2):146-153. doi:10.1056/NEJMoa1505126 26760085

[28] Brown LD, Cai TT, DasGupta A Interval estimation for a binomial proportion. Stat Sci. 2001;16:101-133. doi:10.1214/ss/1009213286

[29] Grant AM; EU Hernia Trialists Collaboration Open mesh versus non-mesh repair of groin hernia: meta-analysis of randomised trials based on individual patient data [corrected]. Hernia. 2002;6(3):130-136. doi:10.1007/s10029-002-0073-1 12209302

[30] Allegranzi B, Bagheri Nejad S, Combescure C, Burden of endemic health-care-associated infection in developing countries: systematic review and meta-analysis. Lancet. 2011;377(9761):228-241. doi:10.1016/S0140-6736(10)61458-4 21146207

[31] Miserez M, Peeters E, Aufenacker T, Update with level 1 studies of the European Hernia Society guidelines on the treatment of inguinal hernia in adult patients. Hernia. 2014;18(2):151-163. doi:10.1007/s10029-014-1236-6 24647885

[32] Nordin P, Zetterström H, Gunnarsson U, Nilsson E Local, regional, or general anaesthesia in groin hernia repair: multicentre randomised trial. Lancet. 2003;362(9387):853-858. doi:10.1016/S0140-6736(03)14339-5 13678971

[33] Löfgren J, Matovu A, Wladis A, Cost-effectiveness of groin hernia repair from a randomized clinical trial comparing commercial versus low-cost mesh in a low-income country. Br J Surg. 2017;104(6):695-703. doi:10.1002/bjs.10483 28206682

[34] Nilsson H, Stylianidis G, Haapamäki M, Nilsson E, Nordin P Mortality after groin hernia surgery. Ann Surg. 2007;245(4):656-660. doi:10.1097/01.sla.0000251364.32698.4b 17414617 PMC1877035

[35] Hoyler M, Hagander L, Gillies R, Surgical care by non-surgeons in low-income and middle-income countries: a systematic review. Lancet. 2015;385(suppl 2):S42. doi:10.1016/S0140-6736(15)60837-6 26313091

