# Supplementary material for: Outcomes After Elective Inguinal Hernia Repair Performed by Associate Clinicians vs Medical Doctors in Sierra Leone: A Randomized Clinical Trial
Source: JAMA Netw Open. 2021 Jan 11;4(1):e2032681. doi: 10.1001/jamanetworkopen.2020.32681 (PMC7801936; doi:10.1001/jamanetworkopen.2020.32681)
Supplement: Supplement 1. — Trial Protocol [file jamanetwopen-e2032681-s001.pdf]

Inguinal hernia surgery in Sierra Leone; an implementation study of low cost anterior mesh repair performed by medical doctors and non-physician clinicians

1. Introduction

Global surgery

Five billion people do not have access to quality surgical services worldwide.(1) The unmet need for surgery is largest in sub-Saharan Africa (SSA) and South Asia.(2) Previously, surgery has been thought to be too resource intensive to be prioritised in poor countries, but simple surgical procedures are recently demonstrated very cost-effective.(3,4) Surgical conditions account for 28-32% of the global burden of disease, but have only recently been understood to be of public health significance.(2,5) The opportunities for research and work to improve surgical service delivery in low-income countries are extensive and much needed.

Global surgery is a field of study, research, practice and advocacy. Its aim is to achieve equity in surgical service delivery for all people worldwide.(6) Conditions like inguinal hernia, that result in a significant burden of disease, can only be successfully treated by surgery.(7) Surgical service provision requires functioning health care systems and infrastructure. Therefore, focusing on a condition like inguinal hernia that is treatable at first level hospitals has the potential to improve the delivery of surgical care in particular, but also act as an enabler for hospital health system strengthening in general.(8)

Inguinal hernia – epidemiology and mosquito nets

Inguinal hernia is a common surgical condition with an estimated 20 million hernia cases repaired every year worldwide,(9) and it is one of the most commonly performed surgical procedures in sub-Saharan Africa.(10,11)

However, 200 million patients living with inguinal hernia do not receive necessary surgical care. In Uganda where the annual hernia repair rate is 17 per 100,000 people, the met need for inguinal hernia surgery is less than 1% per year.(12)

In a systematic review the minimum total need for hernia repair in sub-Saharan Africa was 204 per 100,000, of which 175 per 100,000 were unmet.(13) The high number of inguinal hernias in Sub-Saharan Africa that go unrepaired results in a higher number of complications. In a study in East Africa, over 175 in 100,000 needed surgery for inguinal hernia and there were 30 strangulated hernia per 100,000 each year.(14)

Using mesh to achieve a tension-free inguinal hernia repair significantly reduces the risk of hernia recurrence and has been the preferred method in high-income countries for decades.(15) However, in resource constrained settings, most inguinal hernias are repaired using tissue techniques, which has significantly poorer outcomes compared to tension free mesh techniques. A commercial mesh costs over US$100 and more than doubles the cost of a hernia repair in Uganda.(16) The cost of a commercial mesh in Sierra Leone is also around US$100. A recently published double-blinded, randomized clinical trial carried out in Uganda shows that a mosquito net, which comes at a fraction of the cost of a commercial net, is a safe and effective alternative for elective inguinal hernia repair.(17) Mosquito net costs $0.01, and the cost of sterilization is $1.46.(18) In order to prove benefit for patients living in low-resource settings, this finding needs to be translated into practice.

Task-shifting

Task-shifting with non-surgeons is practiced in many African countries, depending on national policies .(19) The World Health Organisation (WHO) defines task-shifting as “the rational redistribution of tasks among health workforce teams from highly qualified workers to health workers with shorter training and fewer qualifications.”(20) In sub-Saharan Africa, non-surgeon physicians and Non-Physician Clinicians (NPCs) commonly perform inguinal hernia repair. NPCs are mid-level health care providers who receive training to perform duties that medical doctors would normally do. In this case, they are trained to undertake a selected number of surgical procedures. Though there is some data to support the safety of inguinal hernia repair by NPCs in Tanzania, further research is needed to support this practice for mesh repair.(21) NPCs in Sierra Leone are called Community Health Officers (CHOs), and some of them perform surgical procedures. According to recent data collected as part of the post-Ebola recovery process in March 2016 there are 648 CHOs in Sierra Leone.(22)

A nationwide inventory of surgical procedures and providers carried out in Sierra Leone in 2012 showed that there were 164 surgical providers. Of these, 14 (8.4%) were CHOs and they carried out 6.8% of the total national volume of surgery.(23)

Sierra Leone

Sierra Leone is a small country in West Africa, with an estimated population of just above 6.3 million people.(24) The country has been badly affected by a 10-year civil war between 1992 and 2002 and more recently experienced a devastating outbreak of Ebola.

In 2008 there were only 10 qualified surgeons catering for the whole of the Sierra Leonean population.(25) The Ebola outbreak has had a further impact on numbers of qualified doctors, as 11 out of the country’s 125 clinically practicing medical doctors died during the outbreak .(26) The shortage of doctors correlates with a huge unmet need for healthcare in general and surgery in particular. The unmet need for surgery in Sierra Leone has recently been documented to be 92.1%.(27) However, this calculation was done prior to the Ebola outbreak and the unmet need is likely higher now.

In 2012, 22.4% of all surgeries performed in Sierra Leone were hernia repairs. The prevalence of inguinal hernias in Sierra Leone has been estimated to be about 7%, while Ghana and Tanzania have documented prevalence’s of 10.8% and 12%, respectively .(28)(29)(30) Thus, the actual prevalence of inguinal hernia in Sierra Leone is likely to be in the range between 7 -12%.

Since 2011, CapaCare, a Norwegian NGO, has been collaborating with the Ministry of Health and Sanitation and the United Nations Population Fund in Sierra Leone to provide a Surgical Training Programme for non-surgeon doctors and CHOs. As of May 2016, the programme has trained 9 clinicians and another 26 are currently in training. The CHOs that complete the training are called Surgical Assistant CHOs. Second after caesarean sections, inguinal hernia repair is the most commonly performed surgical procedure by the trainees and the graduates of the programme. (31)

Financial burden

Sierra Leone ranks as 181 in the UN Human Development Index and 56.6% of the population live below the income poverty line (less than $1.25 per day).(32)

Healthcare for pregnant, lactating women and children under 5 has been delivered free since 2010 in Sierra Leone. Besides free treatment for HIV, TB and Malaria, all other healthcare costs are paid by the patient, which is considered the main barrier to access healthcare.(33) Catastrophic health expenditure is defined in relation to a household's capacity to pay and occurs when the health costs exceed 40% of the total household expenditure.(34)(35)

Hernia repair in Sierra Leone costs between US$ 50-75 and represents a vast financial burden that forces many into catastrophic health expenditure. Patients with hernias may also experience disability and potential loss of earnings. A 2013 cluster survey from Sierra Leone recorded that 20% of patients were unable to work due to their untreated hernias.(28) Furthermore, delay in seeking treatment for hernia is associated with higher morbidity and mortality and higher cost of emergency surgery in the event of strangulation.(36)

This project based in Sierra Leone will investigate how non-surgeon medical officers and surgical assistant CHOs can be trained to perform mesh hernia repair under local anaesthesia. The study will also compare outcomes between these two staff categories in order to detect any significant differences that will have to be taken into consideration for large-scale implementation of this hernia repair method in Sierra Leone and beyond. A cost-effectiveness analysis will also be undertaken. The Sierra Leone site contributes to a multicentre study on inguinal hernia repair in sub-Saharan Africa. It is part of a collaboration between multiple partnerships and builds on several years of research in African environments.(11,12,16,17)(23,26,27)

2.1 Benefit for patients and health services

A low cost material can be used instead of an expensive mesh in groin hernia repair. To meet the vast need for surgery, task-shifting is an approach that is already practised in Sierra Leone.(23) However, the safety and effectiveness of this practice in groin hernia repair, specifically using a synthetic mesh has not been evaluated sufficiently. This study will do that and the results will be used to implement training initiatives, write national treatment guidelines and to inform policy makers, funders, NGOs and governments. The patients participating in the study will benefit directly as they will have their hernias repaired. The potential benefit for many millions of groin hernia patients living in resource scarce settings worldwide is considerable.

Integral to this study is the training of doctors and CHOs in mesh hernia repair, thereby directly improving standards in groin hernia repair. In addition, it is likely that this study will improve surgical quality and the function of the first level hospitals in general.

3. Topics and objectives

The purpose of this project is to assess low cost mesh inguinal hernia repair in Sierra Leone. The specific aims are to:

1. Evaluate feasibility and effectiveness of implementation of inguinal hernia repair using low cost mesh
2. Assess whether non-physician clinicians and doctors can deliver comparable outcomes in inguinal hernia repair using low cost mesh.
3. Analyse cost-effectiveness of inguinal hernia mesh repair in Sierra Leone.

These aims will be addressed by a single blinded, randomized clinical trial.

4. Feasibility

The participants of this research team contribute with valuable and unique knowledge and experiences that ensure the feasibility of the project.

The PhD student, Thomas Ashley, is a Sierra Leonean medical doctor with experience from working in a variety of hospitals in Sierra Leone, with over 6 months at both of the study sites. He completed the CapaCare surgical training programme in 2013 and is currently undertaking postgraduate surgical residency training in Ghana with the West African College of Surgeons.

The Norwegian collaborators have extensive experience of clinical work and research, both previous and current in Sierra Leone. The Swedish collaborators, carried out a similar randomized clinical trial on groin hernia surgery in Uganda with follow up rates of over 95% one year postoperatively.(17)

4.1. Design, methodology and analysis

Theory

A low cost mosquito mesh can be used instead of an expensive commercial mesh to electively repair inguinal hernias in adult males.(17) An implementation study of the low cost mesh repair is necessary to guide the introduction of the technique on large scale in resource-constrained settings.

To increase access to surgical services, task-shifting with non-surgeons and NPCs is already practiced in several sub-Saharan African countries.(19) A study comparing these staff cadres is necessary to determine the safety and efficacy of task-shifting of mosquito net mesh repair.

Method

Study design: Single-blinded, randomised clinical trial

Study population: The project will be undertaken in two hospitals in northern Sierra Leone – Kamakwie Wesleyan Hospital and Masanga Hospital, both longstanding partner hospitals in CapaCare's Surgical Training Programme.

Sample size calculation: *Statistical rationale:* Based on a non inferiority design the assumptions are as follows: 80% power, 5% significance level, 5% non-inferiority limit and expected success rate of 98% in both arms results in a sample size of 97 individuals in each group. Correcting for an expected 15% loss to follow up we adjust the sample size to 114 in each group, or 228 in total.

Patient selection:

Inclusion criteria: 1. Age > 18 years. 2. Reducible, primary inguinal hernia. 3. The patient accepts participation and is capable of giving informed consent.

Exclusion criteria: 1. Female. 2. Recurrent hernia. 3. Femoral hernia. 4. Ongoing anticoagulant medication. 5. Current obvious drug abuse. 6. ASA group 3-6

Surgical material and technique: The anterior tension-free mesh repair according to Lichtenstein will be performed under local anaesthesia using a sterilized low cost mesh. The mesh is made of lightweight polyethylene. Prior to the operation, the mesh will be cut into 10x15 cm strips. Thereafter the mesh will be cleaned using water and a mild detergent, packed and autoclaved at 121 degrees Celsius for 20 minutes. This has been shown to ensure adequate sterility and minimal changes to the mesh.(37) Preoperatively, the patients will be given one prophylactic dose of 1.5 grams of Flucloxacillin orally. Local pre-operative routines for elective hernia surgery will be

followed. The World Health Organisation (WHO) surgical safety checklist for surgery will be used for all surgeries.

Surgical training: Certified consultant surgeons (the trainers) will train the surgical-assistant CHOs and the medical officers (the trainees) in tension-free mesh hernia repair. Only trainees currently performing and proficient in open tissue inguinal hernia repair will be trained. Trainees will attend a two-week training course including lectures on mesh repair and local anesthesia administration for inguinal hernia repair. Trainees will observe five mesh inguinal hernia repairs and perform three mesh repairs under supervision by the trainers. After the training, two trainers will independently assess trainee operative skills using a checklist based on the American Board of Surgery Operative Performance Assessment Form for open inguinal hernia.(38) Upon successful completion of the assessment, enrollment of patients into the study will begin. A minimum of five surgical assistants CHO and five medical officers will be included.

Data collection: Data will be collected on the previous educational and surgical experience of each hernia surgery trainee as well as the specifics of their training in mesh hernia repair for this study (number of procedures observed, performed under supervision, and practical test scores). Patient interviews including medical history, the EuroQol 5D (EQ5D) and the Inguinal Pain Questionnaire (IPQ) will be done preoperatively, at follow up two weeks and one year after surgery. Physical examination will be undertaken preoperatively in conjunction with the interviews to verify that the patients meet the inclusion criteria,and also at two weeks and one year postoperatively in other to detect post operative complications and recurrences. Expenses associated with the training and the surgeries will be recorded.

Data analysis: Surgical trainee data will be analysed descriptively. Primary endpoints for patient data include hernia recurrence. Secondary endpoints include postoperative complications, chronic pain and patient satisfaction. The outcomes following surgery performed by medical doctors and CHOs will be compared. Results from Sierra Leone will be compared with results from the other collaborating centres. When comparing data, two sample t-tests will be used for continuous variables and Pearson's Chi-square or Fisher exact test will be used for counts. A p-value < 0.05 will be considered statistically significant.

Cost effectiveness will be expressed as USD per Disability Adjusted Life Year (DALY) averted and USD per Quality Adjusted Life Year (QALY) gained. The EuroQol questionnaire will be used to calculate QALYs gained (before surgery compared to one year postoperatively).(39) The Inguinal Pain Questionnaire results will be used to calculate DALYs gained (before surgery compared to one year postoperatively).(40)

The cost analysis will be done from the care provider's perspective. It will include staff time, material consumption in relation to the surgery as well as hospital overhead costs and capital costs.

Disability weights for the DALY calculation will be achieved by converting the results from the seven-level IPQ into the three-level disability weights for abdominopelvic as outlined in the Global Burden of Disease study 2010.(41) The disability weight one year after the surgery will be subtracted from the disability weight before the surgery. DALYs averted will be calculated by multiplying the disability weight difference with the remaining life expectancy of the patient

according to the WHO life table for Sierra Leone.(42) The mean DALYs averted will be presented.

QALYs gained will be calculated by translating the results from the EQ5D questionnaires into index values. The difference in the index value before and one year after the surgery will be multiplied with the remaining life expectancy of the patient. The mean QALYs gained will be presented.

In line with the Global Burden of Disease study 2010, no time and age weighting will be used.(41) A sensitivity analysis will be undertaken to assess the robustness of the assumptions.

Equipment and Personnel

The study requires functioning operation theatres and surgical equipment. To ensure that high volumes of surgery can be performed, we will provide the needed surgical instruments, medicines and materials not available at the selected study sites. Depending on availability, we might need to invest in one reliable autoclave per participating hospital.

The principal investigator based in Sierra Leone will be responsible for co-ordination of all activities of the study, supported by a core team. The principal investigator together with CapaCare will organise training and ensure that additional capacity needs at the chosen hospitals are feasible for the hernia surgery. The core team will be responsible for data collection from recruitment of patients to follow up.

4.2. Organization and collaboration

This project based in Sierra Leone contributes to the multicentre project on Inguinal hernia repair in sub-Saharan Africa. It is part of a collaboration built up of four bilateral partnerships (Sierra Leone-Norway, Uganda-Sweden, US-Ghana/Tanzania).

Individuals

NORWAY

Principal supervisor is Professor **Arne Wibe** MD, PhD, FASCRS. As research coordinator of the Norwegian Rectal Cancer Group he is one of the major international premise providers on colorectal cancer. As a colorectal surgeon, he is attached to the Dept. of Cancer Research and Molecular Medicine of the Norwegian University of Science and Technology (NTNU) and St. Olavs University Hospital in Trondheim. Dr. Wibe is main supervisor on two ongoing PhD projects in Sierra Leone.

Håkon Bolkan, MD, general surgeon at St. Olavs Hospital, and co-founder of CapaCare. He is a research fellow at NTNU exploring surgical capacity in Sierra Leone. He has a vast international experience with Médecins Sans Frontières (MSF), among others as the President for 2.5 years. He led a team of Norwegian healthcare workers for the UN Mission for Ebola Emergency Response (UNMEER) during the outbreak in Sierra Leone and he is the principal investigator on a joint research initiative between NTNU and Karolinska Institute, looking at the effects of Ebola on the Sierra Leonean health care system. Dr Bolkan will be co-supervisor.

Alex Van Duinen, MD, surgeon in training at St. Olavs Hospital, research fellow at NTNU comparing outcomes after caesarean sections done by Surgical Assitants CHO’s and Medical Doctors in Sierra Leonean district hospitals. Dr. Van Duinen has worked in Sierra Leone for 2,5 years as a medical doctor in-charge at one of the study sites, Masanga Hospital.

SIERRA LEONE

Dr. Foday Sahr, MBChB, PhD, Consultant microbiologist, Head of 34 Military Hospital, Freetown, Former acting principal of College of Medicine and Allied Health Sciences, University of Sierra Leone. He has extensive experience in research and he will be local co-supervisor for this PhD.

Dr Thaim B. Kamara, MBChB, FWACS is a consultant urologist and Medical Director of the main national tertiary surgical hospital and Senior Lecturer and Head of the Department of Surgery at College of Medicine and Allied Health Sciences. He was one of the 25 Commissioners of the Lancet Commission on global surgery and member of WHO's Global Initiative for Emergency and Essential Surgical Care.

SWEDEN

Jenny Löfgren, MD, PhD, KI Joint supervisor, co-ordination of study activities together with Jessica Beard.

Andreas Wladis, MD, Surgeon, Associate Professor of Surgery, joint supervisor, overall project leader of multicentre study together with Pär Nordin.

Pär Nordin, UmU, MD, Surgeon, Associate Professor of surgery. Collaborator. Project leader together with Andreas Wladis.

US-GHANA/TANZANIA

Jessica Beard, MD, MPH is a chief resident in general surgery at University of California San Francisco. She has studied outcomes after surgery done by NPCs in Tanzanian district hospitals and was the lead author on the chapter, "Hernia and hydrocele" in the Essential Surgery volume of the Disease Control Priorities in Developing Countries, 3rd Edition by the World Bank. Dr. Beard will be collaborator.

4.3. Plan for implementation

The results from the study will be used to influence national policy both on hernia repair and on the training of CHOs and non-specialist medical doctors in Sierra Leone.

The results of the study may be presented at national, regional and international level.

- Global: Bi-annual meeting of the World Health Organization's Global Initiative for Essential and Emergency Surgical Care and Alliance for Surgery and Anaesthesia Presence (ASAP)
- Africa: West African College of Surgeons annual conference
- Sierra Leone: Annual biomedical conference in Sierra Leone
- Norway: NTNU Annual Global Health Day
- UK: Royal College of Surgeons, Association of Surgeons of Great Britain and Ireland Annual Conference

5. Ethics

Ethical approval will be obtained from the medical ethics committee in Sierra Leone and Norway. Patients will be included in the study after giving informed consent, information will be provided verbally and in a written form in local languages. The surgeries will be offered free of charge, and transportation costs will be compensated. Participants will be encouraged to contact the research team in case of any complications or questions during the follow up period.

6. References (Project participants in bold)

1. Weiser TG, Regenbogen SE, Thompson KD, et al. An estimation of the global volume of surgery: a modelling strategy based on available data. Lancet Lond Engl. 2008 Jul 12;372(9633):139–44.
2. Meara JG, Leather AJM, Hagander L, et al. Global Surgery 2030: evidence and solutions for achieving health, welfare, and economic development. The Lancet. 2015 Aug;386(9993):569–624.
3. Chao TE, Sharma K, Mandigo M, et al. Cost-effectiveness of surgery and its policy implications for global health: a systematic review and analysis. Lancet Glob Health. 2014 Jun;2(6):e334–45.
4. Grimes CE, Henry JA, Maraka J, et al. Cost-effectiveness of surgery in low- and middle-income countries: a systematic review. World J Surg. 2014 Jan;38(1):252–63.
5. Bae JY, Groen RS, Kushner AL. Surgery as a public health intervention: common misconceptions versus the truth. Bull World Health Organ. 2011 Jun 1;89(6):394.
6. Dare AJ, Grimes CE, Gillies R, et al. Global surgery: defining an emerging global health field. Lancet Lond Engl. 2014 Dec 20;384(9961):2245–7.
7. Jamison DT, Breman JG, Measham AR, et al., editors. Disease Control Priorities in Developing Countries [Internet]. 2nd ed. Washington (DC): World Bank; 2006 [cited 2016 Jun 6]. Available from: http://www.ncbi.nlm.nih.gov/books/NBK11728/
8. Editors TPlM. A Crucial Role for Surgery in Reaching the UN Millennium Development Goals. PLOS Med. 2008 Aug 26;5(8):e182.
9. **Beard JH**, Ohene-Yeboah M, Devries CR, Schecter WP. Hernia and Hydrocele. In: Debas HT, Donkor P, Gawande A, Jamison DT, Kruk ME, Mock CN, editors. Essential Surgery: Disease Control Priorities, Third Edition (Volume 1) [Internet]. Washington (DC): The International Bank for Reconstruction and Development / The World Bank; 2015 [cited 2016 Jun 6]. Available from: http://www.ncbi.nlm.nih.gov/books/NBK333501/
10. Galukande M, Schreeb J von, **Wladis A**, et al. Essential Surgery at the District Hospital: A Retrospective Descriptive Analysis in Three African Countries. PLOS Med. 2010 Mar 9;7(3):e1000243.
11. **Löfgren J**, Kadobera D, Forsberg BC, Mulowooza J, **Wladis A, Nordin P.** District-level surgery in Uganda: Indications, interventions and perioperative mortality. Surgery. 2015 Jul;158(1):7–16.
12. **Löfgren J**, Makumbi F, Galiwango E, **Nordin P**, Ibingira C, Forsberg BC, et al. Prevalence of treated and untreated groin hernia in eastern Uganda. Br J Surg. 2014 May;101(6):728–34.
13. Grimes CE, Law RSL, Borgstein ES, et al. Systematic review of met and unmet need of surgical disease in rural sub-Saharan Africa. World J Surg. 2012 Jan;36(1):8–23.
14. Nordberg EM. Incidence and estimated need of caesarean section, inguinal hernia repair, and operation for strangulated hernia in rural Africa. Br Med J Clin Res Ed. 1984 Jul 14;289(6437):92–3.
15. Scott NW, McCormack K, Graham P, et al. Open mesh versus non-mesh for repair of femoral and inguinal hernia. Cochrane Database Syst Rev. 2002;(4):CD002197.
16. **Löfgren J**, Mulowooza J, **Nordin P**, **Wladis A**, Forsberg BC. Cost of surgery in a low-income setting in eastern Uganda. Surgery. 2015 Jun;157(6):983–91.
17. **Löfgren J, Nordin P**, Ibingira C, Matovu A, Galiwango E, **Wladis A**. A Randomized Trial of Low-Cost Mesh in Groin Hernia Repair. N Engl J Med. 2016 Jan 14;374(2):146–53.
18. Clarke MG, Oppong C, Simmermacher R, et al. The use of sterilised polyester mosquito net mesh for inguinal hernia repair in Ghana. Hernia J Hernias Abdom Wall Surg. 2009 Apr;13(2):155–9.
19. Federspiel F, Mukhopadhyay S, Milsom P, et al. Global surgical and anaesthetic task shifting: a systematic literature review and survey. Lancet Lond Engl. 2015 Apr 27;385 Suppl 2:S46.
20. WHO | Task shifting: global recommendations and guidelines [Internet]. WHO. [cited 2016 Jun 1]. Available from: http://www.who.int/workforcealliance/knowledge/resources/taskshifting_guidelines/en/
21. **Beard JH,** Oresanya LB, Akoko L, et al. Surgical Task-Shifting in a Low-Resource Setting: Outcomes After Major Surgery Performed by Nonphysician Clinicians in Tanzania. World J Surg. 2014 Jan 10;38(6):1398–404.
22. Human Resources for Health Summit June 2016, Sierra Leone Briefing Document. 2016.
23. **Bolkan HA**, Hagander L, von Schreeb J, Bash-Taqi D, **Kamara TB**, Salvesen Ø, et al. Who is performing surgery in low-income settings: a countrywide inventory of the surgical workforce distribution and scope of practice in Sierra Leone. The Lancet. 2015 Apr;385:S44.

24. Sierra Leone | Data [Internet]. [cited 2016 Jun 7]. Available from: http://data.worldbank.org/country/sierra-leone
25. Kingham T, **Kamara TB**, Cherian MN, et al. Quantifying surgical capacity in sierra leone: A guide for improving surgical care. Arch Surg. 2009 Feb 1;144(2):122–7.
26. **Bolkan HA**, Bash-Taqi DA, Samai M, et al. Ebola and Indirect Effects on Health Service Function in Sierra Leone. PLoS Curr [Internet]. 2014 [cited 2016 Jun 3]; Available from: http://currents.plos.org/outbreaks/?p=49852
27. **Bolkan HA**, Schreeb JV, Samai MM, Bash-Taqi DA, **Kamara TB**, Salvesen Ø, et al. Met and unmet needs for surgery in Sierra Leone: A comprehensive, retrospective, countrywide survey from all health care facilities performing operations in 2012. Surgery. 2015 Jun 1;157(6):992–1001.
28. Patel HD, Groen RS, **Kamara TB**, et al. An estimate of hernia prevalence in Sierra Leone from a nationwide community survey. Hernia. 2014 Apr;18(2):297–303.
29. Ohene-Yeboah M, **Beard JH**, Frimpong-Twumasi B, et al. Prevalence of Inguinal Hernia in Adult Men in the Ashanti Region of Ghana. World J Surg. 2015 Nov 17;40(4):806–12.
30. **Beard JH,** Oresanya LB, Akoko L, et al. An estimation of inguinal hernia epidemiology adjusted for population age structure in Tanzania. Hernia J Hernias Abdom Wall Surg. 2014 Apr;18(2):289–95.
31. CapaCare Annual Report 2015.
32. Human Development Reports [Internet]. [cited 2016 Jun 7]. Available from: http://hdr.undp.org/en/countries/profiles/SLE
33. Groen RS, Sriram VM, **Kamara TB**, et al. Individual and community perceptions of surgical care in Sierra Leone. Trop Med Int Health TM IH. 2014 Jan;19(1):107–16.
34. Organization WH, others. Designing health financing systems to reduce catastrophic health expenditure. 2005 [cited 2016 Jun 6]; Available from: http://apps.who.int/iris/handle/10665/70005
35. Kawabata K, Xu K, Carrin G. Preventing impoverishment through protection against catastrophic health expenditure. Bull World Health Organ. 2002;80(8):612–612.
36. Mbah N. Morbidity and mortality associated with inguinal hernia in Northwestern Nigeria. West Afr J Med. 2007 Dec;26(4):288–92.
37. Stephenson BM, Kingsnorth AN. Safety and sterilization of mosquito net mesh for humanitarian inguinal hernioplasty. World J Surg. 2011 Sep;35(9):1957–60.
38. Resident Assessments | American Board of Surgery [Internet]. [cited 2016 Jun 6]. Available from: http://www.absurgery.org/default.jsp?certgsqe_resassess
39. EuroQol - About EQ-5D [Internet]. [cited 2016 Jun 6]. Available from: http://www.euroqol.org/about-eq-5d.html
40. Fränneby U, Gunnarsson U, Andersson M, Heuman R, **Nordin P**, Nyrén O, et al. Validation of an Inguinal Pain Questionnaire for assessment of chronic pain after groin hernia repair. Br J Surg. 2007 Dec 28;95(4):488–93.
41. Murray CJL, Vos T, Lozano R, et al. Disability-adjusted life years (DALYs) for 291 diseases and injuries in 21 regions, 1990-2010: a systematic analysis for the Global Burden of Disease Study 2010. Lancet Lond Engl. 2012 Dec 15;380(9859):2197–223.
42. GHO | By category | Life tables by country - Sierra Leone [Internet]. WHO. [cited 2016 Jun 6]. Available from: http://apps.who.int/gho/data/?theme=main&vid=61480
