# Supplementary material for: Outcomes After Elective Inguinal Hernia Repair Performed by Associate Clinicians vs Medical Doctors in Sierra Leone: A Randomized Clinical Trial
Source: JAMA Netw Open. 2021 Jan 11;4(1):e2032681. doi: 10.1001/jamanetworkopen.2020.32681 (PMC7801936; doi:10.1001/jamanetworkopen.2020.32681)
Supplement: Supplement 2. — eTable 1. Trainee and Trainer Characteristics eTable 2. Summary of Mortalities at 1 Year [file jamanetwopen-e2032681-s002.pdf]

Supplemental Online Content

Ashley T, Ashley H, Wladis A, et al. Outcomes after elective inguinal hernia repair performed by associate clinicians vs medical doctors in Sierra Leone: a randomized clinical trial. *JAMA Netw Open*. 2021;4(1):e2032681. doi:10.1001/jamanetworkopen.2020.32681

eTable 1. Trainee and Trainer Characteristics
eTable 2. Summary of Mortalities at 1 Year

This supplemental material has been provided by the authors to give readers additional information about their work.

© 2021 Ashley T et al. *JAMA Network Open.*

eTable 1. Trainee and Trainer Characteristics

**Trainees – Medical Doctors and Associate Clinicians**						
	**Age at study start**	**Sex**	**Years in current profession**	**Camp (1-3)**	**Procedures performed in study**	**Recurrences N (%)**
**MD 1**	34	F	6	1	27	0 (0)
**MD 2**	30	M	1	2	20	1 (5·0)
**MD 3**	32	M	4	2, 3	21	2 (9·5)
**MD 4**	29	M	4	2, 3	18	3 (16·7)
**MD 5**	27	F	1	2, 3	16	1 (6·3)
**AC 1**	35	M	4	1, 3	18	0 (0)
**AC 2**	29	F	1	1	13	0 (0)
**AC 3**	39	M	4	2, 3	16	0 (0)
**AC 4**	34	M	3	2, 3	19	0 (0)
**AC 5**	33	M	1	2, 3	21	0 (0)
**AC 6**	38	M	4	2, 3	20	1 (5·0)

MD=medical doctor
AC=associate clinician

**Trainers**				
	**Country of residence**	**Academic title**	**Professional title**	**Camp (1-3)**
**1**	Sweden	Professor	Senior Consultant General Surgeon	1
**2**	Sweden	Associate Professor	Senior Consultant General Surgeon	1
**3**	Ghana	Associate Professor	Senior Consultant General Surgeon	2
**4**	Norway	PhD	Senior Consultant General Surgeon	2
**5**	Lithuania	PhD	Senior Consultant General Surgeon	3

© 2021 Ashley T et al. *JAMA Network Open.*

eTable 2. Summary of Mortalities at 1 Year

**Age**	**Time between operation and death**	**Informant**	**Details**
80	8.5 months	Daughter	Patient had asthma and hypertension. No more information available.
54	9 months	Brother	“Chronic internal pain” after fall from palm tree prior to the operation.
58	9 months	Wife and brother	Abdominal pain apparently unrelated to the hernia, admitted to hospital, died 2 days later. No surgery done.
50	3 months	Friend	Snake bite
36	Uncertain	Relatives in village	Fell from palm tree

© 2021 Ashley T et al. *JAMA Network Open.*
