# Supplementary material for: Outcomes After Elective Inguinal Hernia Repair Performed by Associate Clinicians vs Medical Doctors in Sierra Leone: A Randomized Clinical Trial
Source: JAMA Netw Open. 2021 Jan 11;4(1):e2032681. doi: 10.1001/jamanetworkopen.2020.32681 (PMC7801936; doi:10.1001/jamanetworkopen.2020.32681)
Supplement: Supplement 3. — Data Sharing Statement [file jamanetwopen-e2032681-s003.pdf]

Data Sharing Statement

Ashley. Outcomes After Elective Inguinal Hernia Repair Performed by Associate Clinicians vs Medical Doctors in Sierra Leone. *JAMA Netw Open*. Published January 11, 2021. doi:10.1001/jamanetworkopen.2020.32681

Data
Data available: No

Additional Information
Explanation for why data not available: Data may be shared upon request. Making data publicly available was not part of the ethical approval.
